# Making fingers and words count in a cognitive robot

**DOI:** 10.3389/fnbeh.2014.00013

**Published:** 2014-02-03

**Authors:** Vivian M. De La Cruz, Alessandro Di Nuovo, Santo Di Nuovo, Angelo Cangelosi

**Affiliations:** ^1^Dipartimento di Scienze Cognitive, della Formazione e degli Studi Culturali, Università degli Studi di MessinaMessina, Italy; ^2^Centre for Robotics and Neural Systems, School of Computing and Mathematics, Plymouth UniversityPlymouth, UK; ^3^Facoltà di Ingegneria e Architettura, Università degli Studi di Enna “Kore”Enna, Italy; ^4^Dipartimento dei Scienze della Formazione, Università degli Studi di CataniaCatania, Italy; ^5^Unità operativa di Psicologia, IRCCS Oasi Maria SS di TroinaEnna, Italy

**Keywords:** embodied cognition, developmental robotics, finger counting, number words, number cognition

## Abstract

Evidence from developmental as well as neuroscientific studies suggest that finger counting activity plays an important role in the acquisition of numerical skills in children. It has been claimed that this skill helps in building motor-based representations of number that continue to influence number processing well into adulthood, facilitating the emergence of number concepts from sensorimotor experience through a bottom-up process. The act of counting also involves the acquisition and use of a verbal number system of which number words are the basic building blocks. Using a Cognitive Developmental Robotics paradigm we present results of a modeling experiment on whether finger counting and the association of number words (or tags) to fingers, could serve to bootstrap the representation of number in a cognitive robot, enabling it to perform basic numerical operations such as addition. The cognitive architecture of the robot is based on artificial neural networks, which enable the robot to learn both sensorimotor skills (finger counting) and linguistic skills (using number words). The results obtained in our experiments show that learning the number words in sequence along with finger configurations helps the fast building of the initial representation of number in the robot. Number knowledge, is instead, not as efficiently developed when number words are learned out of sequence without finger counting. Furthermore, the internal representations of the finger configurations themselves, developed by the robot as a result of the experiments, sustain the execution of basic arithmetic operations, something consistent with evidence coming from developmental research with children. The model and experiments demonstrate the importance of sensorimotor skill learning in robots for the acquisition of abstract knowledge such as numbers.

## Introduction

Whether finger counting is an essential stage in the development of the cognition of number is still highly debated, though strong evidence exists on the positive contribution of sensorimotor skills and representation in numerical cognition. A growing number of researchers, consider finger counting an important tool children (as well as adults) use across a variety of cultures in the development of numerical cognition (e.g., Andres et al., 2008; Di Luca and Pesenti, 2011). Consideration of the links between counting and the emergence of number concepts is not new. Piaget (1952) for example, considered the linking of numbers to objects as being an important characteristic of the sensorimotor stage of cognitive development, possibly being one of a series of prerequisites for the child's construction of the concept of number. Quite recently, however, the topic of finger based number knowledge has seen a surge of new interest, especially from embodied cognition perspectives (for a recent special issue on the topic see Fischer et al., 2012). Finger counting has generally been assumed to be important to the acquisition of a mature counting system (e.g., Gelman and Gallistel, 1978; Fuson et al., 1982; Butterworth, 2005) as well as instrumental to the development of children's arithmetic abilities (e.g., Fuson and Kwon, 1992). It has been hypothesized that this capability helps children to acquire a variety of principles proposed as being fundamental to the development of a counting system (Gelman and Gallistel, 1978) such as: the acquisition of the one-to-one correspondence principle (i.e., when counting only one word is assigned to each object) through the tagging or assignment of one number word to each item, the assimilation of the stable order principle (i.e., when counting, number words are always assigned in the same order) and the cardinality principle (i.e., the last number word uttered when counting, is the total number of objects in a set). More recent studies have reported an association between finger gnosis (the ability to mentally represent one's fingers) and mathematical abilities (Noël, 2005; Costa et al., 2011), and found finger training helpful in improving the performance of children with weak numerical skills (Gracia-Bafalluy and Noël, 2008). Evidence such as this supports the view that finger representations play a special role in number cognition, and might serve as a basic building block in the child's unfolding capacity to mentally manipulate abstract numerical information.

That a close link might exist between finger counting strategies and patterns, and that they may influence the mental representation and processing of number, has also been suggested by evidence coming from neuroimaging studies. For example, studies using fMRI on adult subjects to investigate aspects of embodied theories of cognition have found intrinsic functional links between finger counting and number processing. Cortical motor activity is evoked by Arabic digits and number words, which reflects particular individual finger counting habits (i.e., whether when counting small digits subjects started with their right or left hand) (Tschentscher et al., 2012). These results have been interpreted in several different ways by the authors of this study, one interpretation invoking a shared neural network for number processing and planning of finger movements, which would include parietal cortical areas, the precentral gyrus and the primary motor cortex, in which number perception might very well elicit the sub-threshold tendency to move associated fingers. Another interpretation used by these authors, to explain how the association between numbers, number words and individual finger counting movements might have come about in their subjects, during their individual development of numerical skills in childhood, would be predicted by a Hebbian learning approach to semantic circuits (Pulvermüller, 1999). The prediction is, that due to the fact that children often use their fingers when counting and solving simple counting problems, a correlation between the neuronal activation for the processing of numbers and the movement of fingers is established. A number of neuroimaging studies done in the last decade, using both PET and fMRI, had already found activation of part of the left precentral gyrus (where hand movements are represented) when subjects were asked to engage in numerical tasks such as addition (e.g., Pesenti et al., 2000), subtraction (Rueckert et al., 1996) and multiplication (Dehaene et al., 1996), leading some authors to suggest that the activation of the left precentral gyrus, along with the inferior parietal cortex, might be evidence of a finger moving network that might, in turn, be reflecting a trace of a finger counting strategy (Sato and Lalain, 2008).

The act of counting often involves the acquisition and use of a verbal number system, of which, number words are the basic building blocks. Number words are highly frequent in child directed speech, but their meanings are acquired slowly, with effort and in stages. Wynn (1992) has argued, in fact, that the developing knowledge of the meanings of counting words is a central part of the process of understanding the counting system. Though children as young as 6 months can discriminate between set sizes (Xu et al., 2005), and children 1 and 2 years of age show to be good at reciting the count sequence (Fuson, 1988), as well as capable of recognizing number words as designators of quantity (Bloom and Wynn, 1997), their difficulty seems to lie in understanding *how* specific words match to specific quantities. One proposal is that the syntax of number words as well as the contexts, in which they appear, might be serving as cues that help children bootstrap this process early (e.g., Gleitman, 1990; Wynn, 1992; Bloom and Wynn, 1997), but finger counting may also very well be serving as an early entry point to this understanding.

Other evidence coming from developmental as well as neurocognitive studies, in keeping with what has been found in neuroimaging studies, suggest that finger counting activity, helps build motor-based representations of number that continue to influence number processing well into adulthood, suggesting that abstract cognition may be rooted in bodily experience (Domahs et al., 2010). In fact, these motor-based representations have been argued to facilitate the emergence of number concepts from sensorimotor experience through a bottom-up process (Andres et al., 2008). In our view, finger counting, can also be seen as a means by which direct sensory experience with the body can serve the purpose of *grounding* number as well as number words initially as low level labels, that later serve as the basis for the acquisition of new higher level symbols from the combination of already grounded ones, something known as grounding transfer (e.g., Harnad, 1990; Cangelosi and Riga, 2006). The grounding approach has also been useful for the modeling of the acquisition of words for objects (Morse et al., 2010; Tikhanoff et al., 2011) and for actions (Marocco et al., 2010; Stramandoli et al., 2012) as well as for numbers (Ruciński et al., 2011, 2012).

In sum, while finger counting may not be strictly necessary for children to get on their way to the cognition of number, there is evidence that it does seem to help the learning process, serving as a bridge between possibly innate abilities to perceive and respond to numerosity (e.g., Butterworth, 2005) and the development of the capacity to mentally represent and process number as well as linguistic number related concepts (Lafay et al., 2013). Not much work using robotics has attempted to build on this.

A number of connectionist models have simulated different aspects of number learning. Ma and Hirai (1989) for example, studied how children learn to count using an associative memory network model, which mimicked three phenomena proposed by Fuson et al. (1982), to be present in the acquisition of counting by children [i.e., number word sequence produced by children dividable into three distinct portions: conventional, stable nonconventional, and unstable; irregular number words (e.g., “fifteen”) omitted more often than regular ones (“fourteen,” “sixteen”; initially number word sequence is in recitation form)].

Other models yet, have focused on the identification of the number of objects in a visual scene as a result of learning. Dehaene and Changeux (1993), for example, using a system consisting of three modules (an input retina, an intermediate topological map of object locations, and a map of detectors) created a numerosity detector. The system was able to simulate the distance effect in counting, by which performance increases with increasing numerical distance between two discriminated quantities. More recently, Ahmad et al. (2002), explored quantification abilities and how they might arise in development, using a multi neural net approach, that combined supervised and un-supervised nets and learning techniques in order to simulate subitization (phenomenon by which subjects appear to produce immediate quantification judgments, usually involving up to 4 objects, without the need to count them) and counting. They used a combined and modular approach, providing a simulation of different cognitive abilities that might be involved in the cognition of number, (each of which would have their own evolutionary history in the brain), and is in keeping with Dehaene's triple code model (2000). Rajapakse et al. (2005), targeted aspects of language related to number such as linguistic quantifiers. Using a hybrid artificial vision connectionist architecture, they ground linguistic quantifiers such as *few*, *several*, *many*, in perception, taking into consideration contextual factors. Their model, after being trained and tested with experimental data using a dual-route neural network, is able to count objects (fish) in visual scenes and select the quantifier that best describes the scene. Even more recently, Ruciński et al. (2011) using a cognitive robotics paradigm, have explored embodied aspects of mathematical cognition such as the interactions between numbers and space, reproducing three psychological phenomena connected with number processing, namely size and distance effects, the SNARC effect and the Posner-SNARC effect. The same group in another work using the same paradigm (Ruciński et al., 2012), instead focused on counting, and in particular, on the contribution of counting gestures such as pointing. These models, however, did not consider the role of finger counting in numerical abilities.

In this paper, using a Cognitive Developmental Robotics paradigm (Asada et al., 2009; Cangelosi and Schlesinger, 2014) we present results of an exploration on whether finger counting and the association of number words (or tags) to the fingers, could serve to bootstrap the representation of number in a cognitive robot enabling it to perform basic numerical operations, such as addition.

## Materials and methods

The robotic model used for the experiments is a computer simulation model of the iCub humanoid robot (Tikhanoff et al., 2008, 2011). The iCub is an open-source humanoid robot platform designed to facilitate cognitive developmental robotics research as detailed in (Metta et al., 2010). At the current state the iCub platform is a child-like humanoid robot 1,05m tall, with 53 degrees of freedom (DoF) distributed in the head, arms, hands and legs. The simulated iCub has been designed to reproduce, as accurately as possible, the physics and the dynamics of the physical iCub. The simulator allows the creation of realistic physical scenarios in which the robot can interact with a virtual environment. Physical constraints and interactions that occur between the environment and the robot are simulated using a software library that provides an accurate simulation of rigid body dynamics and collisions. One of the most advanced parts of the iCub is the hand, that comprises 9 DoF, for a total of 18 DoF, and it is the result of a design that optimized the level of integration of the hand in the overall robot to meet the child-like project specifications in terms of dimensions, dexterity and sensorization. Details on the iCub hand can be found in (Schmitz et al., 2010).

**Figure 1:**
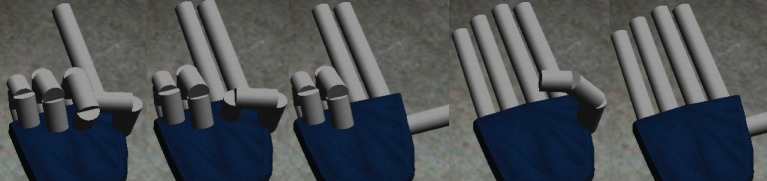
**Number representation with the right hand fingers of the iCub**. From left to right: one, two, three, four and five.

In this work we focus on the fingers, that means we use 7 DoF for each hand, distributed as follows: 2 DoF for thumb, index and middle fingers, but only one for controlling the ring and pinky fingers, that are “glued” together. Because of the limitation with the last two fingers the finger representation of numbers with the right hand is as in Figure 1. Numbers from six to ten are represented by adding left hand fingers with all the right hand fingers open (e.g., six is five right hand plus one left hand). In this work, we suppose that the robot is right handed.

The iCub is not provided with ears, so the auditory input (i.e., the number words) was recorded from a child's voice using a standard microphone and stored as a WAVEform audio file format at 22 KHz with lossless compression. From the waveform, we extracted the mel-frequency cepstral coefficients (MFCC) to represent each number word from one to ten, using the Slaney's auditory toolbox 2.0 for MATLAB (1998). MFCC technique combines an auditory filter-bank with a cosine transform to give a rate representation roughly similar to the auditory system (Davis and Mermelstein, 1980).

**Figure 2:**
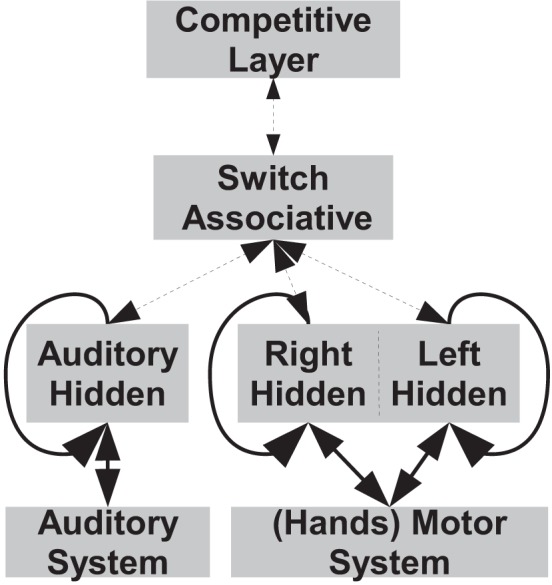
**Schematic of the Robot's Cognitive System**. In the lower part are the units of the motor controller/memory and of the auditory sub-systems, they are directly connected with the robotic platform. In the upper part there are the units with abstract functions that are the switch/associative network and the competitive layer classifier. Bold links indicate a full (one-to-all) connection between each layer, while dotted links are direct (one-to-one) connections. Note that the system's external inputs coincide with the outputs, indeed proprioceptive information from motor and auditory systems is an input for the system during the training phase, while it is the control output when the system is operating.

Figure 2 presents the architecture of the robot's cognitive system, in which the different units and their connections are presented in a schematic form. The lower part of the implemented neural system is directly connected with the robotic platform, and can be summarized in: (i) the motor controller/memory (Motor System and Right/Left Layers), that is able to plan finger movements by setting the finger joints' angles and to memorize the finger number sequence; (ii) an auditory memory (auditory system and auditory layer), that is able to memorize the number words sequence. Upper part of Figure 2 presents the inner units that are responsible for abstract functions (i.e., not directly connected with the robot), they are the switch/associative layer, that allows the two lower systems to cooperate in order to perform other functions, and the competitive layer classifier we implemented to test the quality of the number learning. After supervised training, it is able to represent the correspondence between numbers from 1 to 10 and the internal representations (i.e., hidden layer activations and/or cepstral coefficients). The role of the competitive layer classifier is to simulate the final processing of the numbers, after a number is correctly classified into its class, the appropriate action can be started, e.g., the production of the corresponding word, of a symbol, the manipulation of an object and so on.

The motor controller/memory was designed using two different RNNs in order to model lateralization when processing numbers, as shown by (Tschentscher et al., 2012). In this way, the network that controls the left hand will be switched off when low numbers (1–5) are processed. The two RNNs that compose the motor controller/memory were trained separately, i.e., with different random weight initialization. Note that the motor controller is implemented by two different RNNs, trained separately, but that we refer to as a single unit. The use of RNNs to learn to count was investigated by Rodriguez et al. (1999), they explored the capabilities of recurrent networks in the task of learning to predict the next character in a simple deterministic context-free language, in order to provide a more detailed understanding of how dynamics can be harnessed to solve language problems.

The artificial neural networks were implemented using the Matlab Neural Network Toolbox 8.0, the supervised training algorithm for all networks was Levenberg-Marquardt algorithm (LMA), one of the fastest and widely used optimization algorithms that can be applied to artificial neural networks (Hagan and Menhaj, 1994). The LMA interpolates between the Gauss–Newton algorithm (GNA) and the method of gradient descent. The LMA is more robust than the GNA, which means that in many cases it finds a solution even if it starts far from the final minimum. Like the quasi-Newton methods, the LMA was designed to approach second-order training speed without having to compute the Hessian matrix. When the performance function has the form of a sum of squares (as is typical in training feed-forward networks), then the Hessian matrix can be approximated as ***H*** = ***J****^*T*^****J*** and the gradient can be computed as ***g*** = ***J****^*T*^****e*** where ***J*** is the Jacobian matrix that contains first derivatives of the network errors with respect to the weights and biases, and ***e*** is a vector of network errors. In our implementation, the error ***e*** is calculated as the average of the squared errors of outputs. The Jacobian matrix can be computed through a standard backpropagation technique (see Hagan and Menhaj, 1994), that is much less complex than computing the Hessian matrix. The LMA uses this approximation to the Hessian matrix in the following Newton-like update:

$$\Delta x={\left[J{\left(x\right)}^{T}J(x)+\mu I\right]}^{-1}J{\left(x\right)}^{T}e(x)$$

when the scalar μ is zero, this is just Newton's method, using the approximate Hessian matrix. When μ is large, this becomes gradient descent with a small step size. Newton's method is faster and more accurate near an error minimum, so the aim is to shift toward Newton's method as quickly as possible. Thus, μ is decreased after each successful step (reduction in performance function) and is increased only when a tentative step would increase the performance function. In this way, the performance function is always reduced at each iteration of the algorithm. In our experiments the initial value of μ was 0.001, increase factor was 10 while decrease factor was 0.1, maximum μ was 10^10^. The number of iterations (or epochs) of the algorithm was variable because we adopted as stop criterion a minimum performance gradient of 10^−7^ or a maximum of 1000 epochs.

The derivative function of the RNN networks was the back-propagation through time (Rumelhart et al., 1986), that is a gradient based technique that begins by unfolding the recurrent neural network through time into feed-forward neural networks, so that the training then proceeds in a manner similar to training a feed-forward neural network with classic back-propagation, except that each epoch must run through the observations in sequential order.

The competitive layer classifier is implemented using the *softmax* transfer function that gives as output the probability/likelihood of each classification. Naturally, it ensures all of the output values are between 0 and 1, and that their sum is 1. The *softmax* function used is a follows:

$$softmax(q,i)=\frac{e^{q_{i}}}{\sum_{j=1}^{n}e^{q_{j}}}$$

where the vector ***q*** is the net input to a *softmax* node, and *n* is the number of nodes in the *softmax* layer.

The architecture of the hidden layers of RNNs was chosen after a performance test, in which after 100 runs with varying number of hidden neurons, the best trade-off solutions were selected in terms of minimization of the error and number of iterations needed to converge. We found that 10 neurons was not surprisingly the ideal solution, this because 10 is also the number of different states to represent. Furthermore, in our preliminary experiments we also found that the pure linear transfer functions for the hidden layers were more effective than the usual sigmoid. We chose not to use a bias or set them to zero for the RNN. Due to these choices, when the networks are not active, i.e., all activations are zero, they can be activated by incepting the activation values to the respective neurons in order to start counting from a specific number.

In addition to the main blocks, an associative network is included in the system to initiate the computation of the system and to implement the number manipulation. Indeed, after the RNNs have learned the number sequence, the switch is needed to stop the counting and to redirect the signals to the competitive classifier for the processing of the result.

**Figure 3:**
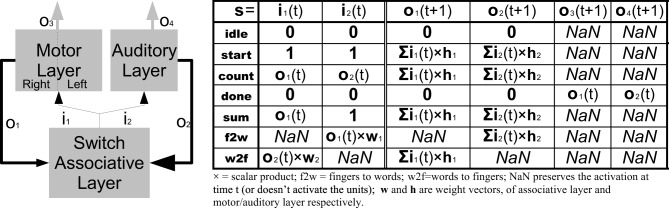
**Details of the Switch/Associative Layer**. The table on the right summarizes the outputs according to the different states. In practice the layer operates as a recursive feedback with the possibility to start and reset the motor/auditory layers and to derive the activations of one layer from the ones of the other. Bold lines indicate a full weighted connection, while normal lines are single connections. For simplicity hidden units of the two RNNs of the motor system are represented with one block.

Figure 3 shows the details of the switch/associative layer that, once the two systems have learned to count, allows them to operate and communicate with each other. In particular, the unit is responsible for starting the counting by initializing all the hidden units to 1, and redirecting the hidden unit activation to the competitive classifier when the counting is finished. Furthermore, this unit is crucial in the development for the acquisition of the ability to add numbers, because it can reset one of the two networks to make it count the new operand, and it lets the other continue as a buffer memory. Finally, thanks to the associative connections between the two layers (with weights **w**_1_ and **w**_2_ in Figure 3) there are other two states that allow inputting a specific number representation starting from another: from fingers to words and vice versa. These states will be studied in more detail in the number manipulation experiments. All states are reported in the table on the left of Figure 3.

As can be seen from the switch/state table in Figure 3 we set to 1 the initial state of all the hidden layers' neurons in order to start the sequence. Vice versa if the initial state is set to 0, there is no activation because RNNs do not have bias in the hidden layer.

Table 1 presents the actual finger joint positions for the ten number representations plus the rest (zero) position. A high value of the joint position represents the finger when it is closed, while low values indicate the finger is open. Note that because of element collision and tendon limitations the actual values are not the ideal ones (i.e., 90, 180, 220 when finger is closed, 0 when open).

**Table 1:** **Actual finger joint positions according to number representation**.

**Fingers**	**Rest/zero**	**One**	**Two**	**Three**	**Four**	**Five**	**Six**	**Seven**	**Eight**	**Nine**	**Ten**
Thumb right	90.0	90.0	90.0	10.9	88.9	11.1	11.1	11.1	11.1	11.1	11.1
	180.0	180.0	180.0	2.1	88.8	1.2	1.2	1.2	1.2	1.2	1.2
Index right	90.0	1.1	0.0	0.0	0.0	0.0	0.0	0.0	0.0	0.0	0.0
	180.0	2.2	0.0	0.0	0.0	0.0	0.0	0.0	0.0	0.0	0.0
Middle right	90.0	90.0	1.1	0.0	0.0	0.0	0.0	0.0	0.0	0.0	0.0
	180.0	180.0	2.2	0.0	0.0	0.0	0.0	0.0	0.0	0.0	0.0
Ring and pinky right	220.0	220.0	220.0	220.0	3.0	0.0	0.0	0.0	0.0	0.0	0.0
Thumb left	90.0	90.0	90.0	90.0	90.0	90.0	90.0	90.0	11.0	89.0	11.0
	180.0	180.0	180.0	180.0	180.0	180.0	180.0	180.0	2.2	88.9	1.2
Index left	90.0	90.0	90.0	90.0	90.0	90.0	1.0	0.0	0.0	0.0	0.0
	180.0	180.0	180.0	180.0	180.0	180.0	2.1	0.0	0.0	0.0	0.0
Middle left	90.0	90.0	90.0	90.0	90.0	90.0	90.0	1.0	0.0	0.0	0.0
	180.0	180.0	180.0	180.0	180.0	180.0	180.0	2.1	0.0	0.0	0.0
Ring and pinky left	220.0	220.0	220.0	220.0	220.0	220.0	220.0	220.0	220.0	2.7	0.0

Table 2 reports the MFCCs for the number words extracted from a child voice.

**Table 2:** **Mel Frequency Cepstral Coefficients for number words**.

**MFCC**	**One**	**Two**	**Three**	**Four**	**Five**	**Six**	**Seven**	**Eight**	**Nine**	**Ten**
1	−35.5929	−32.9669	−32.4777	−31.2712	−29.7136	−35.4331	−35.442	−32.0295	−31.2157	−31.9479
2	−1.0919	−1.3581	−1.5224	−1.8495	−1.4493	−1.2686	−1.0689	−1.9539	−1.1709	−2.0959
3	0.4216	1.1045	0.6798	0.0099	−0.6858	0.7221	0.6448	0.6668	0.1402	0.5683
4	−0.1042	0.2708	−0.0635	−0.3179	−0.4566	0.184	0.1756	0.2295	0.7539	−0.2115
5	0.3303	0.0268	0.2202	0.0331	0.507	−0.08	−0.2727	−0.0303	−0.4317	−0.2456
6	−0.1156	0.3903	−0.0071	−0.3468	0.3923	0.0328	0.0277	−0.2201	−0.189	0.1962
7	0.0052	0.1658	−0.0939	0.3523	−0.3028	0.1184	0.7074	0.0011	0.9789	0.2468
8	−0.1069	0.0182	0.0204	0.3312	−0.1998	−0.4751	0.036	−0.0101	0.3488	0.4278
9	−0.1343	−0.1744	0.0419	0.1559	−0.1472	0.0975	0.2879	0.1467	−0.2435	0.5773
10	0.1164	−0.1774	0.0226	−0.0661	−0.1542	0.5202	0.1546	0.1802	0.4552	−0.1427
11	−0.5587	0.3471	−0.0303	−0.0531	0.3098	−0.1306	0.155	0.0578	−0.1662	−0.0612
12	−0.1981	−0.0564	0.1463	0.0979	0.2068	−0.0164	−0.2105	0.2783	−0.2708	−0.0456
13	0.223	−0.0566	−0.0506	0.033	−0.0655	0.0037	−0.1311	−0.0695	−0.176	0.0915

In our experiments, all values in the input/output datasets used in training were pre-processed by dividing them by the maximum absolute value of the series, in order to have them in the range [−1, 1]. This is beneficial for the learning of weights and biases of the artificial neural networks.

## Experiments and results

Using the material and methods presented above, first we studied the part of the cognitive system that learns to count. The results of the training are presented in the subsection “Numbers learning.” As second step, we build on this by developing the capacity of the associative network to control basic operations like the addition of two operands and to derive the number representation of one of the networks from the other (i.e., from fingers to words and vice versa).

### Numbers learning

For this first experiment, the main goal was to test the ability of the proposed cognitive system to learn numbers by comparing the performance of different ways of training the number knowledge of the robot with: (1) the internal representation (hidden units activation) of a given finger sequence, (2) the MFCC coefficients of number words out of sequence, (3) the internal representation of the number words sequence, (4) the internal representation of finger sequences plus the MFCC of number words out of sequence (i.e., learning words while counting); (5) internal representations of the sequences of both fingers and number words together (i.e., learning to count with fingers and words).

To this end, we setup the experiment with the following steps: (i) the motor controller learns the opening of the fingers in a given sequence, in order to later establish a finger counting routine, and creates an internal representation for each step in the sequence by means of the hidden units activations; (ii) MFCCs are extracted from number words; (iii) the auditory memory learns the verbal number words in order from 1 to 10 and creates an internal representation for each word in the sequence. From each learning step, relevant data are collected and stored as datasets for the experimentation, these sequences can be summarized as follows:

1. Internal representations of the finger sequence: 10 values corresponding to the activation values of the hidden units of motor controller/memory network.
2. MFCCs from number words: 13 values, not as part of a sequence.
3. Internal representations of the words sequence: 10 values from hidden units' activations of auditory memory network.
4. Internal motor representations of the finger sequence and MFCCs: a total of 23 values obtained by merging 1 and 2.
5. Internal motor and auditory representations: a total of 20 inputs obtained by merging 1 and 3.

Datasets 4 and 5 are built to model the learning when both fingers and number words are presented together as training input to the cognitive system.

**Figure 4:**
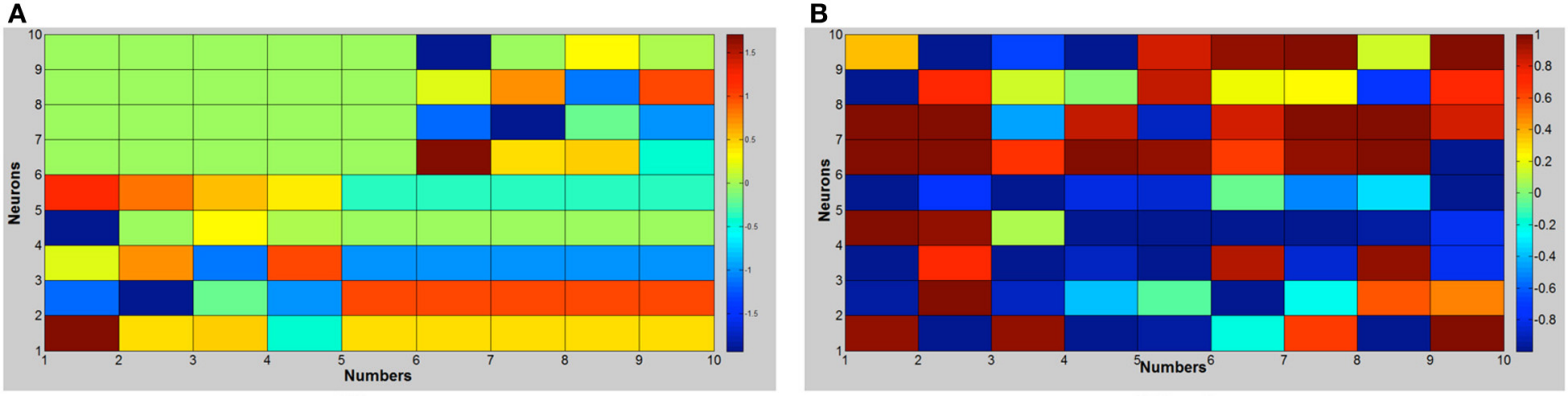
**Hidden units' activation with the number sequence from 1 to 10**. **(A)** RNN trained with finger sequence. **(B)** RNN trained with word sequence.

**Figure 5:**
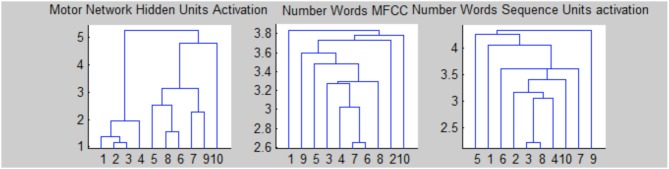
**Optimal leaf-order of hidden units' activations**.

Figure 4 shows the activation values of hidden layers of RNNs: finger sequences on the left and word sequences on the right. Note that we present together the activations of the two RNNs that compose the motor controller/memory network. Motor activations show a lateralization because the network that controls the left hand (neurons 6–10) is switched off, furthermore the units from 1 to 5 remains fixed from the number five on because we suppose that the right hand is open (we reason as if the robot is right handed). Moreover, in Figure 5 we present the dendrogram after the optimal leaf order (Bar-Joseph et al., 2001), that shows how the internal finger representation is more similar to the number sequence, indeed, numbers that are close in the actual sequence are linked together. Meanwhile, the grouping of number words (learned in or out of sequence) is more random, and affects the learning as shown in the classification experiment.

All datasets were used to train the competitive layer classifier to be classified in the ten classes that represent the numbers from 1 to 10. Classification results after 10 epochs of training are presented for each class/number in Table 3. The low number of epochs is, in this case, is imposed in order to study the robot's number learning in the early stages. However, results show that 10 epochs are enough for the LMA to converge.

**Table 3:** **Likelihood of number classes with different training datasets**.

	**Fingers sequence only**		**Number words out of sequence**		**Words sequence only**		**Finger sequence and number words**		**Fingers and words sequences**	
	**Median**	**Std dev**	**Median**	**Std dev**	**Median**	**Std dev**	**Median**	**Std dev**	**Median**	**Std dev**
1	0.779	0.0743	0.3526	0.0218	0.7638	0.0093	0.7355	0.0782	0.9585	0.0108
2	0.662	0.1341	0.2303	0.0083	0.7358	0.0094	0.6584	0.048	0.9621	0.0085
3	0.7015	0.1192	0.0752	0.0043	0.679	0.0145	0.6255	0.0587	0.9459	0.021
4	0.9027	0.1376	0.1777	0.003	0.6414	0.0195	0.7789	0.0677	0.9763	0.1921
5	0.7156	0.0485	0.4448	0.0057	0.6871	0.0116	0.7798	0.0292	0.9148	0.0114
6	0.7574	0.05	0.2411	0.0322	0.6452	0.0161	0.8072	0.0533	0.9202	0.01631
7	0.8226	0.0387	0.2798	0.031	0.6082	0.0308	0.875	0.0229	0.9333	0.01391
8	0.6799	0.0653	0.1499	0.0045	0.6476	0.0198	0.7133	0.0543	0.9125	0.0138
9	0.7854	0.0433	0.3667	0.0058	0.7284	0.0102	0.84	0.0333	0.9443	0.0152
10	0.9069	0.0278	0.2404	0.0058	0.7436	0.0098	0.93	0.0221	0.9708	0.0085
avg	0.7653	0.0738	0.2558	0.0122	0.688	0.0151	0.7743	0.0468	0.9438	0.0311

Table 3 reports the medians and standard deviations of class calculated after 100 runs for each classification training dataset. If we consider “good” classification only, the cases in which the likelihood is greater than 0.5, we can consider the plain words dataset as not adequate to train the network because it fails for all numbers. However, if we consider as successful classification the cases when the class has the greatest likelihood, the only misclassification observed is for the number three. All the other datasets are good and as expected, when finger and word sequences are used together, the cognitive system learns numbers quickly and with a very good likelihood, greater than 90% for all numbers.

We performed a pairwise *t*-test to evaluate the statistical significance of the results reported in Table 3. The *t*-test results confirm that all the differences are statistically significant except for the number three, when finger sequences are compared with word sequences, and the two when finger sequences only are compared with finger sequences and number words.

The “finger sequence and number words” (i.e., dataset 4), shows that associating the number words with the fingers sequence helps to drastically improve the classification performance without needing to learn number words in a sequence. However, to learn number words in sequence helps to additionally improve the classification performance to highest likelihood, if internal representations are associated with motor ones.

**Figure 6:**
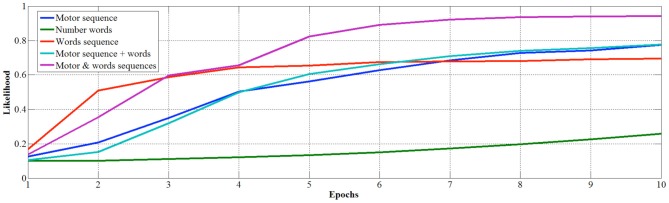
**Average likelihood with number classes with varying epochs**.

**Figure 7:**
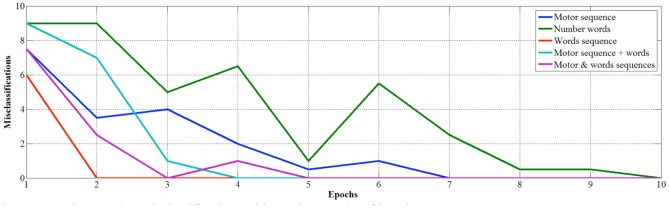
**Median number misclassifications with varying epochs of learning**.

In order to study in more detail the development of learning, we measured the classification performance over the 10 epochs for the competitive layer trained with the different datasets. In this case, performance is evaluated by means of the average likelihood of classification (Figure 6) and median number of misclassifications (Figure 7).

Looking at the developmental results, we once again see that number words learned out of a sequence are the less efficient to learn as there are no misclassifications only after 10 epochs, and the average likelihood is still low (0.256) after ten epochs. Conversely, if number words are learned in sequence and internal representations are used as inputs, the learning is faster in terms of precision of classification (i.e., no errors after just 2 epochs) but the maximum average likelihood, that converges at 0.688, is not as strong as when the learning involves also fingers. Indeed, the finger sequence reaches a higher average likelihood (0.765), but best results are obtained when internal representation of words and fingers are used together as input, in fact the average median likelihood is 0.94 just after 8 epochs.

### Numbers manipulation

Once the number sequences are learned, an interesting feature of the proposed cognitive system is the possibility to easily build up the ability to manipulate numbers with the development of the switch-associative network.

Indeed, this ability can be modeled by extending the capabilities of the associative network from the simple start and stop, to its transferring and mapping to the basic operation of addition.

By transferring, we mean the new mapping of the network's representation derived from the number counted by the other network, when the robot hears the number word “three,” to the correlated finger representation. This can be considered, in a sense, an associative mapping between internal representations. This is implemented by activating a weighted connection between the two networks, which can be learned by applying the LMA to the two-layer network that comprises the hidden units of both networks. This training is quite fast and effective both ways, as an average of 4 iterations (over 100 trials) are needed to reach an average estimation error lower than 10^−15^, which practically does not affect the performance of the classifier, that shows differences in the statistical indicators (mean, median, and standard deviation over 100 trials) lower than 10^−12^ when its inputs are derived by the associative network.

The operation of addition can be seen as a direct development of the concurrent learning of the two recurrent units (motor and auditory). Indeed, if one of the two does the actual counting of the operands, the other can be used as a buffer memory to add the result, when it is done, the final number can be transferred from the buffer to the other unit and then inputted to the final processor (the classifier in our system).

As an example let us consider 2 + 2, the following steps will be taken:

1. The first operand is heard by the auditory system and both networks will count until the corresponding activation of number 2 is reached. This step corresponds to the states of the associative network.
2. The sum operator is recognized so the auditory network is reset, while the first operand remains stored in the motor memory.
3. The second operand is heard, both networks restart to count as in step 1, until the auditory network reaches the activation corresponding to the number 2. In the meantime, the motor network reaches the activation of the number 4.
4. After the auditory network stops, the associative network recognizes that the work is done so the total (4) is incepted from the fingers network to the auditory network thanks to the associative connection.
5. Finally the output of the resulting number (4) is produced for final processing (in our case the classifier).

**Figure 8:**
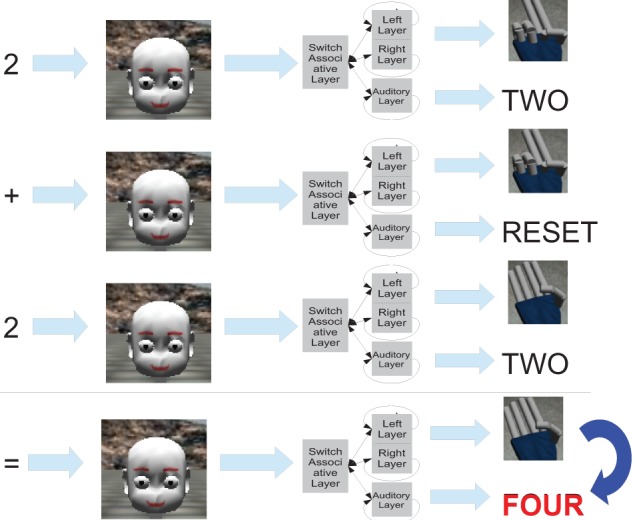
**The picture presents the steps done by the Robot's Cognitive System to perform the addition of two numbers, that in this example are both two**.

The steps are depicted in Figure 8.

## Discussion

The results obtained in our experiments with the iCub child-like robotic platform, show that learning the number words in sequence along with finger configurations helps the fast building of the initial representation of number in the robot. Number knowledge, is instead, not as efficiently developed when number words are learned out of sequence without finger counting. Furthermore, the internal representations of the finger configurations themselves, developed by the robot as a result of the experiments, sustain the execution of basic arithmetic operations, something consistent with evidence coming from developmental research with children.

This does not mean that just learning the counting sequence from one to ten, is enough for children (or our robot), to understand number concepts, but it is the repeated experience using the number word sequence when counting sets of things that is important in the development of numerical understanding (Sarnecka and Carey, 2008; Donlan, 2009). While the use of fingers does not necessarily precede the use of language in the acquisition of a symbolic numerical system (e.g., Nicoladis et al., 2010), what many children seem to be doing initially, in fact, is learning small number word sequences by rote, and later, associations between these small number words and objects in the world (first among which, their readily available fingers). Later on down the developmental path, with the child's early schooling experience, this mapping will also include written representations (or numerals). These written representations, eventually take on the meaning of the spoken number word (Fuson and Kwon, 1992). It is this kind of associative multi-modal learning that we are in a sense reproducing in our model.

Studies focusing on how children acquire abstract words and concepts, have proposed that multiple representational systems involving both sensorimotor as well as linguistic information might be playing a role in conceptual representation (e.g., Louwerse and Jeuniaux, 2010). While the case of the acquisition of number words might be considered as a particular type of abstract word learning, theories such as the LASS theory (Barsalou et al., 2008), according to which both the linguistic system as well as the sensorimotor system (through simulation) are activated in the processing of word meaning to different degrees under different task conditions, and the WAT (Words as Tools) proposal put forth by Borghi and Cimatti (2009) (but also see Borghi et al., 2011, and the special issue of Borghi and Pecher, 2011), have argued and furnished evidence on the synergetic role both language and sensorimotor experience play in the acquisition of abstract concepts, and on how important the modality by which words are learned is. In our model, number words or tags heard repeatedly, when coupled to the experience of moving the fingers, do serve as tools, used in the subsequent manipulation of the quantities they come to represent.

In fact, the internal representations of the finger configurations themselves, found as a result of the experiments, can be considered to be a basis for the building of an embodied number representation in the robot, something in line with embodied and grounded cognition approaches to the study of mathematical cognitive processes. Just as has been found with young children, through the use of finger counting and verbal counting strategies, our model develops finger and word representations that subsequently sustain the robot's learning the basic arithmetic operation of addition. While the experiments done with the model in this work have targeted simple addition, the same model can be easily adapted to implement other operations such as subtraction by training the motor controller/memory with the backward number sequence (from 10 to 1) and then selecting this sequence at the beginning (e.g., by setting all hidden activations to −1), this way the subsequent manipulation will give the result of the subtraction. Future work with the model will implement this.

Another thing we would like to highlight is that, since the hidden layer without any external input does the actual counting, our system is also able to count “mentally” without necessarily producing actions or words. Future work with the model, will explore offline simulation aspects of the motor programs involved in finger based representations of number, or the “mental motor imagery” activated whenever a number and/or number word is encountered, after the robot has learned finger counting and finger calculation early in its training. This direction is stimulated by recent research on the simulation of mental imagery in cognitive systems and robots (see Di Nuovo et al., 2013b), in particular by the successful application of motor imagery models for mental practice in the execution of verbal commands (Di Nuovo et al., 2012) and for performance improvement (Di Nuovo et al., 2013a). The interested reader can find more details about this line of research in a recent special issue (see Di Nuovo et al., 2013b). In recent theoretical accounts of embodied numerosity (e.g., Moeller et al., 2012), something akin to the offline simulation of the motor programs involved in finger based representations of number, has been suggested to take place in children as well as adults. This line of investigation with the model will also compare the representation of embodied numerosity or “manunumeral” representations (Fischer and Brugger, 2011), using different culturally transmitted counting habits or strategies, in order to explore how they might influence the number processing in the robot (e.g., Bender and Beller, 2011; Previtali et al., 2011).

The utility of children's learning finger counting strategies early in their mathematical education continues to be debated in mathematics education research, despite the evidence coming from neurocognitive and psychological studies indicating that it does (for review of debate see Moeller et al., 2011). Our experiments show that in fact, learning to count with the fingers, using verbal tags, can be helpful in the numerical training of a robot as well. While being inspired by the evidence from the studies we have cited in previous sections, our implementation is nonetheless, an abstraction of complex and as of yet not totally understood processes that may underlie the development of numerical cognition. Our results, however, are in line with what has been theoretically claimed in the developmental literature (e.g., Gelman and Gallistel, 1978), that is: that finger counting may be playing a functional role in the acquisition of a variety of principles considered necessary for children to have “under their belts” in order to reach an understanding of number. Examples of these principles and the role of finger counting that are relevant to our present study, and that we think we have simulated at least in part are: finger counting as an aid in the keeping track of the number words while reciting the counting sequence; as it contributing to the induction of the one-to-one correspondence principle by which children are helped by their fingers to coordinate the processes of tagging, or the attribution of a number word to each item; and as facilitating the assimilation of the stable-order principle where numerical labels have to be enumerated in the same order across counting sequences (see also Andres et al., 2008; and Lafay et al., 2013).

The study of mathematical cognitive processes, traditionally considered to be quintessential examples of abstract and symbolic processing, have been assumed to primarily involve the mind rather than the body. Our embodied robot experiments indicate, that aspects of the development of this knowledge can be accounted for not only by way of bodily representations, but also with an artificial network in the place of a mind.

### Conflict of interest statement

The authors declare that the research was conducted in the absence of any commercial or financial relationships that could be construed as a potential conflict of interest.
